# The Correction of Abnormal Conditions of the Blood Relative to Surgical Operations

**Published:** 1905-01

**Authors:** S. C. Emley

**Affiliations:** Wichita, Kan., Late Pathologist Augustana Hospital, Chicago, Ill.


### THE CORRECTION OF ABNORMAL CONDITIONS OF THE BLOOD RELATIVE TO SURGICAL OPERATIONS.

BY S. C. EMLEY, A.B., M.D., WICHITA, KAN.,
Late Pathologist Augustana Hospital, Chicago, Ill.

Frequently the surgeon is called upon to operate on patients who, when they first present themselves, are in no condition to stand an operation on account of deficient quantity of blood or the poorness of its quality. On the other hand, it is desirable that the patient regain his normal condition as soon as possible after operation, whether the abnormal condition of blood is due to the operation or not.

The ideal remedy is that which will restore the normal condition of the blood in the shortest time with the least disturbance to the rest of the body, the digestive system particularly. Less necessary are palatability and cost of the remedy. To determine which of several preparations best fulfilled the above conditions was the purpose of this investigation.

All of the preparations used being recognized as good, Dr. A. J. Ochsner gave me permission to prescribe them as I saw fit to certain of his patients in Augustana Hospital. Only those cases were selected whose appearance indicated the need of a hematinic. As often as possible similar cases were paired off, one patient being given one preparation and the other patient another, and the results compared. The cases were paired according to pathological condition, age, sex, general condition and the condition of the blood as to hemoglobin and erythrocytes at the beginning of treatment. The preparations used were malt with iron and manganese; malt with iron, quinine and strychnine; Blaud's pills, and the preparation known as pepto-mangan (Gude).

After watching the effect of the medication on the patients, and observing the records, it is seen that Blaud's pills acted quickly, but constipated; the malt combinations caused nausea in a few patients, and the malt, manganese and iron combination caused constipation in nearly all. The pepto-mangan given in milk was agreeable to take, and in no case did it cause nausea or constipation. While in two cases the Blaud's pills acted more quickly than pepto-mangan in two similar cases, on the whole the latter gave better and quicker results than any of the others, and at the same time caused no digestive disturbances in any of the cases.

Although the investigation was undertaken for the purpose of finding the best hematinic for surgical cases, it was tried in one case of chlorosis and in several obscure medical cases.

The following table shows the results obtained in all those cases where Gude's preparation was given. One to four drams were given in milk to each case three times a day. The hemoglobin was estimated with Von Fleischel's hemometer, and the erythrocyte count made with the Thoma-Zeiss apparatus. The first blood count was made previous to operation in all surgical cases, and the last a short time before the patient's discharge from the hospital. The second count was never made immediately after the operation because of the temporary derangement due to the anesthetic and the loss of blood :

| NAME. | Age | Diagnosis. | Date. | Erythrocytes per 1 c.c. | Per cent. of Hemoglobin. |
|---|---|---|---|---|---|
| 1. G. N.*...... | 53 | Carcinoma of stomach. | 9 /29 /03 | 2,920,000 | 33 |
| | | | 10 /12 /03 | 3,400,000 | 43 |
| | | | 10 /25 /03 | 3,260,000 | 42 |
| | | | 11 / 8 /04 | 2,520,000 | 36 |
| 2. Mr. L.*..... | 49 | Carcinoma of stomach. | 10 /29 /03 | 2,665,000 | 27 |
| | | | 11 /23 /03 | 2,900,000 | 28 |
| | | | 12 / 5 /03 | 2,540,000 | 27 |
| | | | 12 /19 /03 | 2,300,000 | 26 |
| 3. Miss J...... | 17 | Acute menorrhagia.... | 12 / 4 /03 | 2,310,000 | 36 |
| | | | 12 /20 /03 | 3,565,000 | 44 |
| | | | 12 /27 /03 | 4,160,000 | 49 |
| 4. Mrs. E. K.. | 33 | Menorrhagia.......... | 12 / 7 /03 | 4,340,000 | 44 |
| | | | 1 /10 /04 | 3,565,000 | 64 |
| | | | 1 /18 /04 | 5 100,000 | 82 |
| 5. Mr. S....... | 23 | Neurasthenia(?)........ | 12 /16 /03 | 4,060,000 | 60 |
| | | | 1 / 7 /04 | 4,260,000 | 65 |
| | | | 1 /14 /04 | 4,560,000 | 75 |
| 6. Mr. K...... | 35 | Tuberculosis of mesenteric glands. | 11 /15 /03 | 3,825,000 | 62 |
| | | | 12 /10 /03 | 4,826,000 | 68 |
| | | | 1 / 4 /04 | 4,716,000 | 66 |
| 7. Mrs. F...... | 23 | Pelvic abscess......... | 10 /25 /03 | 4,060,000 | 60 |
| | | | 11 /23 /03 | 5,100,000 | 69 |
| | | | 12 /11 /03 | 4,975,000 | 78 |
| 8. Mrs. A..... | 34 | Pelvic abscess.......... | 12 /10 /03 | 3,195,000 | 53 |
| | | | 12 /29 /03 | 4,293.000 | 58 |
| | | | 1 /11 /04 | 4,560,000 | 78 |
| 9. Miss A. J... | 16 | Chlorosis .............. | 10 /25 /03 | 3,010,000 | 45 |
| | | | 11 /12 /03 | 4,950,000 | 65 |
| | | | 11 /28 /03 | 5,676,000 | 80 |
| 10. Mrs. H..... | 40 | Myoma of uterus....... | 7 /15 /03 | 2,100.000 | 42 |
| | | | 8 /17 /03 | 3,900,000 | 55 |
| | | | 9 /15 /03 | 4,500,000 | 80 |
| 11. Johnny L... | 13 | Tuberculosis of hip..... | 12 / 1 /03 | 2 680,000 | 45 |
| | | | 12 /29 /03 | 3,600.000 | 55 |
| | | | 1 /20 /04 | 4,100.000 | 62 |
| 12. Mr. E. P.... | 21 | Tuberculosis of ankle.. | 10 /29 /03 | 4,310,000 | 66 |
| | | | 11 /10 /03 | 4,850,000 | 71 |
| | | | 1 /23 /04 | 5,166,000 | 75 |
| 13. Johnny F... | 9 | Extensive burn and infection of surface. | 11 / 9 /03 | 3.560,000 | 50 |
| | | | 11 /25 /03 | 3,900,000 | 56 |
| | | | 1 /23 /04 | 4,362,000 | 68 |
| 14. Miss E. B... | 17 | Perforative appendicitis | 11 /25 /03 | 3,600,000 | 55 |
| | | | 12 /26 /03 | 4,000,000 | 65 |
| | | | 1 /22 /04 | 4,250,000 | 69 |
| 15. N. N........ | 29 | Suppurative appendicitis. | 12 /20 /03 | 4,200,000 | 60 |
| | | | 1 / 2 /04 | 4,400,000 | 66 |
| | | | 1 /20 /04 | 5,120,000 | 75 |
| 16. Mr. B...... | 28 | Chronic appendicitis... | 1 / 2 /04 | 3,565,000 | 62 |
| | | | 1 /10 /04 | 4,3[illegible]0,000 | 70 |
| | | | 1 /23 /04 | 4,800,000 | 78 |
| 17. Mr. S....... | 37 | Gangrenous appendicitis. | 10 /10 /03 | 3,300,000 | 45 |
| | | | 10 /27 /03 | 3,350,000 | 45 |
| | | | 11 /27 /03 | 3.010,000 | 40 |
| 18. Miss W. J.. | 29 | Empyema............. | 11 /20 /03 | 2,740,000 | 44 |
| | | | 12 /20 /03 | 3,070,000 | 52 |
| | | | 1 /22 /04 | 3,820,000 | 60 |
| 19. Mr. F....... | 44 | Cholelithiasis chronic appendicitis. | 11 /23 /03 | 3,560,000 | 57 |
| | | | 12 / 4 /03 | 4,100,000 | 68 |
| | | | 1 /12 /04 | 4 640.000 | 78 |

*Incurable.

In the nineteen cases tabulated there is an average increase of 800,000 erythrocytes and of 14.5 per cent. hemoglobin. This improvement was during forty days on an average. The usual time a patient stays in the hospital is twenty-one days when the case is of ordinary severity from a surgical standpoint. Such cases were placed on tonic treatment and showed rapid improvement, but of such cases only one (Case 16) is noted because it might be urged they would improve equally fast with or without a tonic.

It is seen from the above table that even in the cachexia of carcinoma there is a temporary improvement which shows that in the use of this tonic we are dealing with a powerful hematinic. In Case 17 there was no improvement, the patient dying shortly after the last count. At the autopsy I found a pyogenic abscess in the liver as large as an orange and about 200 c.c. of pus below the right kidney, which explained the retrogression. In all of the other operated cases the improvement was steady and marked, especially in uterine diseases accompanied by loss of blood. In the case of chlorosis (No. 9) the improvement was remarkable, the patient being discharged cured in a little over a month, at which time all the symptoms had disappeared.—*Medical News, September 24, 1904.*

